# Experiences and expectations of receiving volunteer services among home‐based elderly in Chinese urban areas: A qualitative study

**DOI:** 10.1111/hex.13624

**Published:** 2022-10-20

**Authors:** Lei Huang, Fengjian Zhang, Lin Guo, Yuqin Chen, Mingjiao Feng, Yanjie You, Lihua Zhang, Ziyun Jiang, Yilan Liu

**Affiliations:** ^1^ Department of Nursing, Union Hospital, Tongji Medical College, Huazhong University of Science and Technology/School of Nursing, Tongji Medical College Huazhong University of Science and Technology Wuhan China; ^2^ School of Sociology Huazhong University of Science and Technology Wuhan China; ^3^ Jiukang Elderly Care Service Corporation Wuhan China

**Keywords:** assistance, expectation, experience, older adults, volunteer service

## Abstract

**Background:**

The various complex needs for assistance among home‐based older adults have increased dramatically. Thus, it would be advantageous to recruit volunteers with medical knowledge and a better understanding to support and assist the elderly living in urban communities.

**Aim:**

This study aimed to explore the experiences and expectations of receiving volunteer services among the home‐based elderly in Chinese urban areas.

**Design, Setting and Participants:**

A descriptive qualitative study was conducted following the Consolidated Criteria for Reporting Qualitative Research (COREQ) guidelines. This study was performed in two communities in Wuhan, Hubei Province. A purposive sampling method, which includes criterion and maximum variation sampling, was used to identify and select a diverse range of participants. Semistructured face‐to‐face interviews with 20 older adults (aged 62–90 years old) were performed. The conventional content analysis method was used for thematic analysis.

**Results:**

Three categories with associated subcategories were identified: experiences of receiving volunteer services including negative and positive experiences; specific needs for volunteer services involving physiological, psychosocial, health‐related behaviours and environmental domains; characteristics of expected volunteer services including availability, formats, recipients, providers and service strategies.

**Conclusions:**

The volunteer services provided to the home‐based elderly were found to be unsatisfactory, and lacking relevance and effectiveness. Due to a lack of family support or difficulty in meeting some high‐level needs, the home‐based elderly expressed a strong demand for volunteer services involving physiological, psychosocial, health‐related behaviours and environmental domains. This finding can provide a basis for developing training plans beneficial to volunteers. Furthermore, the present research clarifies the criteria for selecting volunteers and the necessity of supervising and managing volunteers. Improving the effectiveness and accessibility of urban‐community volunteer service may reduce the burden on care institutions and home caregivers while enhancing the quality of life and well‐being of the elderly.

**Patient or Public Contribution:**

Developing research questions, study design, management and conduct and interpretation of evidence.

## 1 INTRODUCTION

The global population ageing problem has become increasingly serious in recent decades.^1^ Although many countries have taken positive steps to address the ageing crisis, the situation has not improved significantly and the future remains bleak.^2^ The number of people over 60 years of age is expected to double, accounting for 22% of the total global population by 2050.^3^, ^4^ As a country with a large population, China is no exception, and the issue of population ageing is more prominent.^5^ By the end of 2020, the population in China over 60 years of age had reached 264 million, accounting for 18.7% of the total population, with up to 190 million people aged 65 or above.^6^ These increasing numbers have resulted in a slew of social issues, most notably an escalation of the conflict between increased needs for assistance among the elderly and inadequacies in the social system for the provision of services.^7^, ^8^

Unfortunately, the shortage of care service resources leads to the avoidance of care institutions by many older adults, potentially reducing their quality of life.^1^, ^9^ In addition, in some cases the present care institutions and home‐based care service centres fail to meet the diversified needs of the elderly, such as spiritual comfort and health guidance.^10^, ^11^ Furthermore, some older adults are unwilling or unable to seek professional care services due to economic constraints.^12^, ^13^ It has been reported that about 55% of the elderly in China do not have their care needs met.^14^ Therefore, it would be of great benefit to older adults to identify feasible ways in which their needs can be served within the existing social system and resources.

### 1.1 Background

Volunteer service is defined as voluntary and gratuitous public welfare service provided by volunteers, volunteer service organizations and other organizations to society or others.^15^ The forms of volunteer services are diverse, and their nature can be formal, planned and long‐term, or informal, spontaneous and intermittent.^16^ Formal volunteers are those who register in the voluntary service information system designated by the relevant administrative departments in China, receive free accident insurance, participate in training promptly according to the requirements of volunteer service projects, and receive guidance, coordination and supervision from volunteer service organizations while providing services.^15^ Informal volunteers spontaneously organize or participate in voluntary activities without registration. Volunteer service, which has long been associated with positive benefits for older adults, is a potential strategy for coping with caregiver stress.^17^ In particular, under the current situation where the social security system and social welfare are insufficient,^18^ voluntary service undoubtedly plays an important role and bursts out of vitality.^19^ To this end, the ‘14th five‐year plan for healthy aging’ jointly issued by 15 Chinese government departments unequivocally stated the critical need for accelerating the training of social workers and volunteers to serve the health of the elderly.^20^ Volunteer service has multiple advantages. For example, volunteers benefit others, which the elderly prefer.^21^ Volunteers are also eager to learn and master new skills and knowledge to have a multidisciplinary background and are flexible in adapting to individualized needs.^22^ As a consequence, it is critical to actively promote the popularization of volunteer services among the elderly.

Although there have been numerous studies on volunteer service for the home‐based elderly, they have largely focused on volunteer service experience and strategies.^23^, ^24^, ^25^ Gaber and colleagues described their experiences in incorporating community volunteers into the primary care team to assist home‐based older adults. Gau and colleagues described the negative experiences and expectations of community health volunteers in serving the elderly. Liu and colleagues pointed out that volunteerism may also benefit volunteers. Other studies elaborated on the motivations and incentives of volunteers.^26^, ^27^ However, there are very few studies that have explored the feelings and perspectives of the elderly on assistance, nor on whether such assistance is tailored to their actual needs.^28^ A qualitative study in England described the experiences of older adult hoarders being helped by volunteers, but it still focuses on volunteers.^29^ Another study explored the experiences and benefits of receiving volunteer service from a home support programme established to assist older adults but only stated the importance of addressing the social, emotional and mobility needs of the recipients.^30^

These fragmented experiences do not allow for a comprehensive and in‐depth understanding of the need for volunteer services among the home‐based elderly. Furthermore, compared to rural areas, elderly people living in cities have a more difficult life and can easily become disconnected from society.^31^ As a result, it is necessary to investigate the experience and expectations of urban home‐based older adults receiving volunteer service to point out the direction for volunteers to provide assistance services tailored to their needs. The present work will help volunteers clarify their service objectives and improve their service behaviours so that the urban home‐based elderly can benefit more from volunteer services.

### 1.2 Aim

This study aimed to explore the experiences and expectations of receiving volunteer services among home‐based elderly in Chinese urban areas.

## 2 METHODS

### 2.1 Design

This study used a descriptive qualitative research design.^32^ The subjective nature of volunteer services necessitates a thorough investigation. As a result, a qualitative descriptive approach was chosen to clarify the experiences and expectations of home‐based older adults on receiving volunteer services. The author considered and followed the Guidance on Standards for Reporting Qualitative Research to report the study results explicitly and comprehensively (Consolidated Criteria for Reporting Qualitative Research [COREQ]).^33^

### 2.2 Participants

The study was performed in two communities in Wuhan, Hubei Province, in the central part of China. A purposive sampling method, which includes criterion and maximum variation sampling, was used to identify and select diverse participants.^34^ The following inclusion criteria were used: (i) elderly individuals of age 60 years and above; (ii) lived at home; (iii) received assistance from officially registered or informal volunteers after the age of 60. Maximum variation sampling was used to include elderly participants with various difficulties in life (e.g., living alone and suffering from chronic diseases) and in different age groups. We included elderly participants from two communities that were geographically separated. One community is relatively high‐end, while the other is relatively dilapidated, reflecting the different living conditions and socioeconomic status of the elderly. Participants were excluded if they were residing in a long‐term care facility, hospitalized or unable to consent.

### 2.3 Data collection

A total of 20 older adults were interviewed for the project using a semistructured format between October 2021 and December 2021. Data were collected on‐site and conducted in the participant's own home or a separate room in the community activity centre, ensuring privacy and freedom to speak freely. The researcher used a personal information form (including demographic characteristics of older people) and an interview guide during the interviews (including six open‐ended qualitative questions; see Table 1). Only the participant and the researcher were present in the room during the interviews. No one else was present, allowing the participant to express themselves more freely. Each interview lasted between 30 and 60 min. The interview, original manuscript and data analysis were conducted in Chinese. All interviews were recorded using a mobile phone or with a recording pen and transcribed into Chinese using IFLYTEK hearing transcription software. Two bilingual translators translated the selected citations into English to ensure that the meaning remained unchanged.

**Table 1:** Interview questions

1. Can you tell me about something that has bothered you in your daily life? How do you usually solve these problems? 2. Do you obtain any assistance from formal or informal volunteers? If so, can you talk about it in detail? 3. Do you wish to receive any assistance from volunteers? If so, in which areas? If not, what are the reasons for this? 4. What do you expect from the volunteers who will help you? What are the reasons? 5. In what form would you like volunteers to help you? 6. Do you want to add something? Are there any critical aspects we did not talk about so far?

### 2.4 Data analysis

The interview recordings were transcribed verbatim and checked by a trained transcriptionist. The conventional content analysis method was used to sort and analyse the data.^35^ The two researchers encoded the transcribed text data and extracted meaningful coding content to form a rough coding list. After the informal meeting, the two researchers discussed and analysed the coding contents with differences, similarities and correlations to reach a consensus. When the data were close to saturation, the research team interviewed four additional older adults to ensure that the data were saturated and that no new categories appeared. The interview ended after confirming that no further information was obtained. Finally, the research group examined these categories collectively to determine whether they were related and distinct from one another. To facilitate the orderly presentation of the results, the second category was divided based on the problem classification scheme of the Omaha system.^36^ Background information on the participants was obtained by entering the descriptive data into Microsoft Excel (Microsoft Corporation, 2016) and imported into IBM SPSS Version 26 (IBM Corp.) for statistical analysis.

### 2.5 Trustworthiness of the study

The researchers and participants were unfamiliar with each other before data collection to ensure participant interview quality. To ensure the reliability of data collection, the researchers immersed themselves in the data for an extended period and conducted continuous analyses and comparisons. Responses were confirmed several times during the interviews with older respondents. The written records and categories extracted from the analysis were also confirmed by the participants to ensure the credibility and preciseness of the study.

### 2.6 Ethical considerations

The research protocol was approved by the Medical Ethics Committee of Tongji Medical College, Huazhong University of Science and Technology (Ethical review No. S053). Before the interviews, the participants were informed about the purpose and significance of the study, the content of the interviews and the necessity of audio recording. Participants were told that they could withdraw at any time until the end of the interview and that their identities and recordings would be used only for scientific research and would not be disclosed. Participation was voluntary and anonymous, and verbal consent was obtained before the interviews.

## 3 RESULTS

### 3.1 Sample characteristics

A total of 20 home‐based elderly individuals living in urban areas participated in this study. Most participants were female (70.0%), with a mean age of 76.9 years (range: 62–90). All participants were of Han nationality. Most participants were local residents (85.0%) and lived with their families (65.0%) (see Table 2 for details).

**Table 2:** Participant characteristics (*n* = 20)

Characteristics	Number (%)
Gender (female), *n* (%)	14 (70.0)
Age (years) (mean ± SD)	76.90 ± 8.18
Ethnicity	
Han nationality	20 (100.0)
Marital status	
Married, *n* (%)	13 (65.0)
Widowed, *n* (%)	7 (35.0)
Living setting	
With family members, *n* (%)	13 (65.0)
Alone, *n* (%)	7 (35.0)
Migration	
Local residents	17 (85.0)
New immigration	3 (15.0)
Had chronic disease, *n* (%)	15 (75.0)
Individual income (US$/month) (range)	250–900

### 3.2 Summary of qualitative findings

The participants were numbered P1–20 in this study. The data identified three categories and 11 subcategories in the study (see Table 3).

**Table 3:** Overview of main and subcategories

Main category	Subcategory	Codes
Experiences of receiving volunteer services	Negative experiences	Singleness and limitation
		Difficult to really benefit
		Negative impact on actively seeking help
		Lack formal or professional volunteer service
	Positive experiences	Solving existing problems
		Meeting potential demand
Specific needs for volunteer services	Environmental domain	Living environment maintenance
		Establishing a home rescue system
	Physiological domain	Life assistance
		Sleep instruction
	Psychosocial domain	Visiting and chatting
		Psychological support
		Spiritual consolation
		Companionship during special times
		Social contact and intercourse
	Health‐related behaviours domain	Dietary guidance
		Health maintenance
		Disease guidance
		Equipment recommendation
		Accompanying for medical treatment
Characteristics of expected volunteer services	Service availability	Easy to obtain
	Service formats	Remote technology service
		Face‐to‐face service
	Service recipients	Those who need it more
	Service providers	Specific age
		Knowledge background
		Good character
	Service strategies	Building trust
		Targeting demand
		Enhancing family support

### 3.3 Category 1: Experiences of receiving volunteer services

This category presents the experience of receiving volunteer services among elderly individuals living in urban communities to understand their views on the free services received.

#### 3.3.1 Subcategory 1: Positive experiences

A few participants believed that volunteer services could help them to solve some problems in their lives and would be of benefit.

I asked for assistance from the community the last time I went to the hospital alone. They asked volunteers to drive me there. I am extremely grateful to them. (P6)

A few participants mentioned that even if they did not express their needs, some volunteers were able to realize and come up with solutions.

Several volunteers have visited my home. They were concerned that I would become disoriented or fall when I went out, so they hung a sign around my neck. It bears my name, address, and phone number, and I was touched. (P8)

#### 3.3.2 Subcategory 2: Negative experiences

Many participants described that the assistance services they received were single and limited.

A student volunteer once came to my home. He only cleaned the house and chatted with me. (P16)

Most participants were not satisfied with the free services they received from volunteers because the services provided did not meet their actual needs, so it was difficult for them to benefit from them and improve their quality of life.

Volunteers have helped but are not regular and cannot solve some problems. (P19)

Some participants believed their negative experience of receiving volunteer services would discourage them from actively seeking help.

I would not ask for help when there is trouble; there is no place to ask for help. The community does not care at all, and elderly care institutions that provide home care also focus only on benefits; When I am in a bad mood, I will not seek help from others. (P7)

Many participants reported never receiving services from formal or professional volunteers.

The home‐care workers in the care institutions also often engage in volunteer activities, delivering free meals and providing necessary help for us, but no formal or professional volunteers have come. (P2)

### 3.4 Category 2: Specific needs for volunteer services

It was clear from the interviews that the elderly living in urban communities often faced various complex needs in life. Although some of the elderly people whom we interviewed lived with their families, they generally reported that their family members did not have time or energy to provide them with support or help in various ways, including in daily life, despite these issues being easy to solve for their families, let alone the elderly living alone:

My family cannot help me because my daughter‐in‐law needs to take care of my grandson, and my son is not in good health. They cannot care for me, so I must care for myself. However, there are many inconveniences in daily life for that I need help. (P8)

#### 3.4.1 Subcategory 1: Environmental domain

Some participants also mentioned their desire to receive assistance in improving their living environment. Poor living conditions not only bring inconvenience to their lives but also may endanger their lives. For example, fire may be caused by ageing lines:

The toilet is clogged, and the house is flooded with dung; additionally, I am unsure what to do when water pipes and electrical appliances break. I hope someone can come to help repair them regularly. (P12)

I think there is something wrong with the circuit at home. As the circuit is aging. (P13)

Some participants emphasized that they would like to obtain timely responses and assistance from volunteers in case of emergency. It would be better if volunteers had medical knowledge:

I have got heart‐related issues. If there is anything I can say during the day, but what should I do at night? I hope I can contact volunteers who understand medicine. (P3)

When the body is uncomfortable, the children are not around, and it is easy to become nervous. In an emergency, the volunteers could quickly arrive if an emergency pager could be installed at home. (P10)

#### 3.4.2 Subcategory 2: Physiological domain

Some participants expressed the wish that volunteers could assist them in their lives. It was difficult for them to solve seemingly simple problems in everyday life:

I am 90 years old. It is inconvenient to buy vegetables. Sometimes, I want to take a bath, but I am afraid I will fall. (P4)

Many participants mentioned the negative impact of inadequate sleep on their quality of life, hoping that volunteers could assist:

I cannot sleep at night, especially after waking up in the middle of the night and being unable to sleep for hours. Even though I take that sleeping pill daily, I still require assistance for a solution. (P8)

#### 3.4.3 Subcategory 3: Psychosocial domain

Some participants described their desire for volunteers to visit and hoped to have a chat with them:

My husband and I usually watch TV at home or go for a walk, which is very boring. If young people can come and share something new, we would like to listen. (P11)

Some participants hoped to get psychological support to alleviate negative emotions:

Usually, neighbors talk with me, and my lonely mood decreases greatly. I hope volunteers often come to see me and chat. (P14)

Some participants hoped to get spiritual consolation to improve their boring life.

Please teach me how to surf the Internet and buy things using my mobile phone. (P10)

Some participants emphasized that the need for such companionship was stronger during illness or other special times:

When you are ill, it is easy to feel lonely. At this time, you need someone to take care of you and accompany you. (P6)

Some participants expressed a desire for more contact and social interaction with their peers.

We need to build stronger bonds with our neighbors and be able to learn and interact with others. It would be great if volunteers could help us. (P12)

I hope volunteers can organize some activities for the elderly. There is no place to play and no one to play with. (P7)

I usually like painting, but I cannot find anyone with the same interests and hobbies. (P1)

#### 3.4.4 Subcategory 4: Health‐related behaviours domain

Participants described a strong need for the maintenance of health‐related behaviours in general. Several participants mentioned the need for dietary guidance.

It would be great if volunteers could tell us what should we eat and what should we eat less of. (P10)

Some participants hoped to obtain convenient health care services without leaving their homes, such as health monitoring and health‐maintenance consultation, making scientific health decisions on time.

Volunteers can visit the community to help measure blood pressure, blood sugar, and other vital signs and promote health. (P13)

I hope we can obtain some guidance on chronic disease management. (P17)

Many participants hoped to obtain guidance and advice on medication:

I always hope that someone can give me some guidance. For example, I have been taking medicine for a while, and he can help me to see if it has any effect and whether it needs to be mixed. (P2)

A few participants mentioned that some auxiliary equipment might benefit their daily lives and health:

Going up and down stairs is incredibly inconvenient living on an upper floor. Volunteers could suggest some stair‐climbing aids. (P20)

Last time, Jiukang (a geriatric care facility) gave us a mattress that could detect our heart rate and breathing, and we have used it since. I would appreciate more intelligent devices like this in the future. (P5)

Some participants hoped to obtain convenient medical services accompanied by professionals when they needed to visit a doctor:

A person will not go to the hospital. I am unable to locate or make an appointment with a doctor. It is too difficult and requires someone to accompany me. (P9)

### 3.5 Category 3: Characteristics of expected volunteer services

Participants not only described their specific assistance needs but also mentioned some aspects of the expected volunteer services.

#### 3.5.1 Subcategory 1: Service availability

Some participants expressed their concerns about the difficulty in obtaining assistance.

I am an older person with a generation gap with the younger generation. There will be communication issues. Volunteers are not always willing to come. (P15)

#### 3.5.2 Subcategory 2: Service format

Volunteers can provide services for the elderly through door‐to‐door service, which is the ideal form of service:

Send a WeChat message or make a phone call. Of course, they had better come to my home to assist me. (P10)

Volunteers can also help the elderly using technical means. Remote services appear to be easier to implement but they may only be appropriate for certain needs.

Everyone has different requirements. Sometimes, you have to go to the elderly's home based on their personal needs. But if it is a psychological need, it is acceptable to make a phone call. (P17)

#### 3.5.3 Subcategory 3: Service recipient

Some participants stated that they did not require the free services at the present time and hoped to leave these valuable resources to other elderly people, especially those who needed them more.

I do not need anyone to support me, but my classmates need resources to help them. (P17)

#### 3.5.4 Subcategory 4: Service provider

Some participants requested specific volunteers to provide services for them.

I feel older and need the company of younger volunteers. (P18)

Some participants hoped to get assistance from volunteers with medical knowledge.

I hope to get help from volunteers who understand medicine, and It is more practical if neighbors can help each other. (P13)

Furthermore, several participants also believed that volunteers who help the elderly should also have some good qualities, such as love and kindness.

Volunteers should be compassionate, moral, and respectful of the elderly. (P14)

#### 3.5.5 Subcategory 5: Service strategy

Some participants hoped that volunteers could provide targeted help or support to the elderly:

It is hoped that volunteers will first understand our actual situation, whether we require assistance or not, and then decide whether or not to provide it. Otherwise, wasting their time will be pointless. (P12)

A few participants believed that volunteers should provide services in stages to gain the trust and recognition of the elderly:

Nowadays, the elderly are more vigilant, and volunteers should come step by step. If you provide a highly purposeful service, they may be concerned about what you intend to sell. (P10)

A few participants said they would like volunteers to help them get more family support.

Although I have two children, they are usually busy with their work and seldom come. It would be better to let them know they should care more about us and remind them to come and see us more. (P11)

## 4 DISCUSSION

This is the first study to examine the experiences and expectations of receiving volunteer services among home‐based older adults in urban areas. Based on their experiences and expectations, we identified three categories, namely, experiences of receiving volunteer services, specific needs for volunteer services and characteristics of the expected volunteer services.

According to our findings, many participants reported that the volunteer services they received were not satisfactory. Similarly, other studies have described the difficulties experienced by a limited number of volunteers to meet the assistance needs of a large group of elderly people living at home.^37^, ^38^ Therefore, developing public welfare services for the elderly has a long way to go, and volunteer teams in urban communities need to be further expanded. Incentive mechanisms would also be necessary to promote the recruitment and retention of volunteers.^24^, ^25^ In addition, the study also found that it is difficult for the elderly to benefit from volunteer services. This finding is consistent with the results of a Chinese study which found that volunteers had no discernible influence on the life satisfaction of older people living at home.^39^ Interestingly, Cheung and Pan reported that help from volunteers is more significant when the elderly have higher education or lower family income. This may indicate that the experience of being assisted among the home‐based elderly is affected by their characteristics and level of perception. Thus, it may be necessary to direct more volunteers to understand deeply the actual needs of the elderly to provide practical assistance.^13^ Furthermore, negative experiences associated with receiving volunteer services may affect their willingness to ask for help. When faced with all kinds of life troubles, many individuals prefer to suffer silently rather than actively seek assistance from others. Therefore, while advocating voluntary services to these older adults, improving their awareness of active help‐seeking is also particularly important. However, some participants gladly shared their experiences with the researchers, and they were grateful and appreciative of the volunteers, although the volunteers only met their potential needs.

The older adults in this study faced difficulties in almost every domain of their lives, making them one of the most vulnerable groups in society. The predominant message mentioned by participants was the need for assistance in maintaining health‐related behaviours, such as dietary guidance, health counselling and medication guidance. The present findings are supported by previous studies.^1^, ^40^ However, we also found conflicting opinions in the literature regarding the unmet needs of community‐dwelling older adults, which are often nonmedical.^41^, ^42^, ^43^ The reason may be that the older adults in this study, especially individuals with chronic diseases, would prefer to receive professional and irreplaceable assistance from volunteers rather than simple assistance with daily living tasks if given the opportunity.^31^ Besides the identification of the need for comprehensive medical care,^44^ this study also found that many participants hoped that volunteers could recommend useful auxiliary equipment that would be beneficial to them.

Most participants mentioned some life difficulties they hoped that the volunteers could help solve, such as life assistance (e.g., vegetable procurement) and sleep instruction. Although some elderly people lived with their families, they also faced many problems in daily life, either because their children were busy with work or because their spouses also needed care, which is inconsistent with previous studies reporting that family members or caregivers could easily meet the daily life needs of the elderly.^1^, ^45^ Due to financial difficulties or inability to take care of themselves, they did not seek the help of family caregivers or go to nursing. Therefore, they were eager for volunteers to provide them with some necessary help, including help with daily life challenges, instead of their families or caregivers. However, they did not have high expectations of volunteers and rarely mentioned daily care due to the limited support that volunteers can provide. In addition, unlike previous studies,^42^, ^43^ the present study identified additional potential needs for maintaining the living environment, such as the maintenance of ageing circuits, unblocking of pipes and the establishment of an emergency aid system. Although these issues are not difficult to resolve, they can be easily ignored by the family or caregivers, lowering the quality of life of the elderly and even endangering their safety.

Most participants also mentioned needs for psychosocial assistance. The present results, broadly consistent with previous research, also highlighted the need for spiritual comfort and assistance with social interactions among the home‐based elderly.^30^, ^46^ This need for psychosocial services such as chatting and social communication is unsurprising due to the prevalence of loneliness and boredom in the elderly^1^, ^46^ where the isolation of urban life makes them eager for interpersonal interaction.^43^ Therefore, volunteers can help compensate for the lack of social support in this group of elderly people through direct companionship or by assisting them in finding like‐minded partners.^47^ In short, identifying the specific needs of the elderly for volunteer services can provide information for volunteers to improve service quality and re‐examine service behaviour. It can also provide a basis for developing training plans to improve the resource utilization of volunteer services.

Another important category was found to be the characteristics of expected volunteer service, which included availability, formats, recipients, providers and service strategies. Some participants preferred that public welfare assistance be directed toward the elderly in need. They could become volunteers if necessary, implying that volunteer services should prioritize individuals with more pressing needs and encourage the elderly to help each other. Although it is evident that older adults often have difficulty in obtaining volunteer services, this goal can be gradually achieved in the future by improving the visibility and accessibility of voluntary services.^31^ Volunteers should be aware of the diversity and individualization of service formats, such as face‐to‐face interaction or remote technology.^48^ Furthermore, to meet the needs of the elderly, volunteers should improve their personal qualities, broaden their knowledge and insight and form relationships with groups of elderly people that might be in need of their services.^22^ Therefore, before providing assistance services, volunteers should identify the actual needs of the elderly and determine what help or support to offer them based on their advantages. Meanwhile, to maximize service benefits, the urgency of the issue of assistance for the elderly should be assessed to develop a phased service plan. Furthermore, the findings of the study also emphasized the importance of building trust and providing assistance step by step from the perspective of the elderly. Because most elderly people are very cautious and afraid of being cheated, they usually resist strange volunteers and are reluctant to expose their life difficulties. Only when sufficient trust is established can assistance services be more effective. To build a trusting relationship, appropriate self‐disclosure may be beneficial.^29^ Similarly, it is also an effective way to establish a long‐term friendship by gradually making the elderly aware of the empathy and capabilities of the volunteers.^47^ Given the limited role of volunteers, mobilizing family support should also be an essential strategy. While providing real‐time help to the elderly, it is also necessary for volunteers to make an effort to get in touch with their children and communicate effectively concerning difficulties in the lives of the elderly. Meanwhile, volunteers should also focus on developing skills related to talking with the children of the elderly, listening more and blaming less and maintaining timely interaction with them, which will help with the establishment of good relationships. These findings clarify the criteria for selecting volunteers and the necessity of supervising and managing volunteers.

This study has several limitations. First, respondents were asked to recall past experiences, which could be inaccurate or missing essential details. Second, purposive sampling strategies could limit the representativeness of the study population. Moreover, although saturation of participant data was reached, we could not consider all types of older adults, including those with specific difficulties, such as dementia and disability, which could restrict the generalizability of our study findings. However, based on the explorative findings of this study, a further wider‐scale survey might prove insightful.

## 5 CONCLUSION

This study explored the experiences and expectations of receiving volunteer services among the home‐based elderly in Chinese urban areas. The elderly in our study lacked not only the positive experience of receiving voluntary services but also received little free assistance from society. Due to a lack of family support or difficulty in meeting some high‐level needs, the home‐based elderly expressed a strong need for volunteer services involving physiological, psychosocial, health‐related behaviours and environmental domains. These findings can provide a basis for developing training plans for volunteers. This study also clarifies the criteria for the selection of volunteers, implying that they should have extensive background knowledge, good character, selfless dedication and be able to apply useful service strategies. Furthermore, the supervision and management of volunteers should be improved for the elderly to accept and trust volunteers and to ensure that their rights and interests are not violated.

Overall, the findings of this study are of great significance for the selection, training and supervision of volunteers. Improving the effectiveness and accessibility of urban‐community volunteer services may help to reduce the burden on care institutions and home caregivers while also improving the quality of life and well‐being of the elderly.

## AUTHOR CONTRIBUTIONS

*Substantial contributions to conception and design*: Lei Huang, Yilan Liu, Lin Guo, Fengjian Zhang. *Acquisition, analysis and interpretation of data*: Lei Huang, Yuqin Chen, Mingjiao Feng, Yanjie You, Ziyun Jiang. *Drafting the manuscript*: Lei Huang. *Revising it critically for important intellectual content*: Lei Huang. *Final approval of the version to be submitted*: Yilan Liu.

## CONFLICT OF INTEREST

The authors declare no conflict of interest.

## ETHICS STATEMENT

The research protocol has been approved by the medical ethics committee of Tongji Medical College, Huazhong University of science and technology (Ethical review No. S053).

## Supporting information

Supporting information.

## Data Availability

All data are available.
