# Supplementary material for: Experiences and expectations of receiving volunteer services among home‐based elderly in Chinese urban areas: A qualitative study
Source: Health Expect. 2022 Oct 20;25(6):3164–74. doi: 10.1111/hex.13624 (PMC9700141; doi:10.1111/hex.13624)
Supplement: Supplementary file 1 — Supporting information. [file HEX-25--s001.docx]

COREQ Statement—Checklist of items that should be included in reports of qualitative studies

|  | Item No | Recommendation | | Page No |
| --- | --- | --- | --- | --- |
| ****Domain 1: Research team and reflexivity**** | | | | |
| Personal Characteristics | 1 | Interviewer/facilitator | Which author/s conducted the interview or focus group? | The first author |
|  | 2 | Credentials | What were the researcher's credentials? E.g. PhD, MD | PhD candidate |
|  | 3 | Occupation | What was their occupation at the time of the study? | No occupation |
|  | 4 | Gender | Was the researcher male or female? | Male |
|  | 5 | Experience and training | What experience or training did the researcher have? | Trained |
| Relationship with participants | 6 | Relationship established | Was a relationship established prior to study commencement? | 6 |
|  | 7 | Participant knowledge of the interviewer | What did the participants know about the researcher? | 6 |
|  | 8 | Interviewer characteristics | What characteristics were reported about the interviewer/facilitator? e.g. Bias, assumptions, reasons and interests in the research topic | 6-7 |
| ****Domain 2: study design**** | | | | |
| Theoretical framework | 9 | Methodological orientation and Theory | What methodological orientation was stated to underpin the study? | 5 |
| Participant selection | 10 | Sampling | How were participants selected? | 5 |
|  | 11 | Method of approach | How were participants approached? | 5-6 |
|  | 12 | Sample size | How many participants were in the study? | 5 |
|  | 13 | Non-participation | How many people refused to participate or dropped out? Reasons? | *Not applicable* |
| Setting | 14 | Setting of data collection | Where was the data collected? | 5-6 |
|  | 15 | Presence of non-participants | Was anyone else present besides the participants and researchers? | 5-6 |
|  | 16 | Description of sample | What are the important characteristics of the sample? | 7 |
| Data collection | 17 | Interview guide | Were questions, prompts, guides provided by the authors? Was it pilot tested? | 5-6 |
|  | 18 | Repeat interviews | Were repeat interviews carried out? If yes, how many? | *Not applicable* |
|  | 19 | Audio/visual recording | Did the research use audio or visual recording to collect the data? | 5-6 |
|  | 20 | Field notes | Were field notes made during and/or after the interview or focus group? | 5-6 |
|  | 21 | Duration | What was the duration of the interviews or focus group? | 5-6 |
|  | 22 | Data saturation | Was data saturation discussed? | 5-6 |
|  | 23 | Transcripts returned | Were transcripts returned to participants for comment and/or correction? | 5-6 |
| ****Domain 3: analysis and findings**** | | | | |
| Data analysis | 24 | Number of data coders | How many data coders coded the data? | 7 |
|  | 25 | Description of the coding tree | Did authors provide a description of the coding tree? | 7 |
|  | 26 | Derivation of themes | Were themes identified in advance or derived from the data? | 7 |
|  | 27 | Software | What software, if applicable, was used to manage the data? | 6 |
|  | 28 | Participant checking | Did participants provide feedback on the findings? | *6* |
| Reporting | 29 | Quotations presented | Were participant quotations presented to illustrate the themes / findings? Was each quotation identified? | 6 |
|  | 30 | Data and findings consistent | Was there consistency between the data presented and the findings? | 6 |
|  | 31 | Clarity of major themes | Were major themes clearly presented in the findings? | 7-14 |
|  | 32 | Clarity of minor themes | Is there a description of diverse cases or discussion of minor themes? | 14-18 |
